# Optimized prediction of breast cancer tumor microenvironment using MRI-based intratumoral and peritumoral radiomics: a prospective study

**DOI:** 10.3389/fonc.2025.1654508

**Published:** 2025-11-06

**Authors:** Eun Sil Kim, Sungwon Ham, Bo Kyoung Seo, Ji Young Lee, Woong Sun, Minkyu Jeon, Minseok Joo, Seonghoon Park, Shuncong Wang, Boram Lee, Hye Yoon Lee, Min Sun Bae, Kyu Ran Cho, Ok Hee Woo, Sung Eun Song, Soo-Yeon Kim

**Affiliations:** 1 Department of Radiology, Korea University Ansan Hospital, Korea University College of Medicine, Ansan, Republic of Korea; 2 Healthcare Readiness Institute for Unified Korea, Korea University Ansan Hospital, Korea University College of Medicine, Ansan, Republic of Korea; 3 Department of Anatomy, Brain Korea 21 Plus Program for Biomedical Science, Korea University College of Medicine, Seoul, Republic of Korea; 4 Department of Computer Science and Engineering, College of Informatics, Korea University, Seoul, Republic of Korea; 5 Department of Radiology, University of Cambridge, Cambridge, United Kingdom; 6 Division of Breast and Endocrine Surgery, Department of Surgery, Korea University Ansan Hospital, Korea University College of Medicine, Ansan, Republic of Korea; 7 Department of Radiology, Korea University Anam Hospital, Korea University College of Medicine, Seoul, Republic of Korea; 8 Department of Radiology, Korea University Guro Hospital, Korea University College of Medicine, Seoul, Republic of Korea

**Keywords:** radiomics, magnetic resonance imaging (MRI), tumor microenvironment (TME), breast cancer, peritumoral region, artificial intelligence, extracellular matrix (ECM), immune infiltration

## Abstract

**Objective:**

The tumor microenvironment (TME), composed of non-tumor elements such as stromal matrix and immune cells, plays a critical role in tumor progression, metastasis, and treatment response. This study aimed to investigate the association between MRI-based intratumoral and peritumoral radiomic features and the TME components, including extracellular matrix (ECM) and immune cells, in patients with invasive breast cancer.

**Methods:**

In this prospective study, 121 women with histologically confirmed invasive breast cancer underwent pre-treatment multiparametric 3T breast MRI, including T2-weighted, diffusion-weighted imaging (DWI), and dynamic contrast-enhanced T1-weighted sequences (NCT06095414, registered at ClinicalTrials.gov). The dataset was randomly divided into training and testing cohorts in a 7:3 ratio. A total of 16180 radiomic features were extracted from both intratumoral and peritumoral regions. Three-dimensional volume histology with quantitative immunohistochemical staining of ECM and immune cells served as the reference standard for TME assessment. Predictive models were developed using least absolute shrinkage and selection operator regression and evaluated using area under the receiver-operating characteristic curve (AUC). Model performance was compared between intratumoral-only and combined intratumoral–peritumoral features across five MRI sequences.

**Results:**

Models incorporating both intratumoral and peritumoral features significantly outperformed those using intratumoral features alone in predicting TME components (P < 0.01). Among the five sequences, initial and delayed postcontrast T1-weighted images yielded the highest AUCs. For ECM abundance, the AUCs (95% CI) were 0.82 (0.78–0.87) and 0.82 (0.78–0.88) on initial and delayed imaging, respectively. For immune cell abundance, the AUCs were 0.82 (0.77–0.87) and 0.83 (0.78–0.88). Most of the top predictive features were first-order and texture features associated with tissue heterogeneity. Combined models more accurately captured ECM-rich and immunosuppressive TME profiles, characterized by elevated regulatory T cells and reduced cytotoxic T cells, which were associated with poor prognosis.

**Conclusion:**

MRI-based radiomic features from both intratumoral and peritumoral regions are significantly associated with TME components in invasive breast cancer. Contrast-enhanced T1-weighted sequences provided the most robust performance. These findings highlight the potential of MRI-based radiomics as a powerful noninvasive biomarker for characterizing the TME and informing personalized therapeutic strategies, including immunotherapy and ECM-targeted treatments.

## 1 Introduction

Breast cancer is a heterogeneous disease with various biological phenotypes associated with different clinical courses and prognoses (1). The TNM staging system and molecular subtyping are the most important factors in treatment decisions for breast cancer (2, 3). However, large variations in treatment response and outcomes occur even among cases with identical stages or subtypes. The tumor microenvironment (TME) represents one of the key features contributing to tumor behavior and response to treatment. It is composed of various non-tumor cells, including immune cells and the extracellular matrix (ECM) (4, 5). Thus, assessing the status of the TME is crucial for precision medicine in breast cancer, possibly leading to improved treatment outcomes.

Among the components of the TME, an abundant, stiffened, and disorganized ECM mainly composed of collagen, laminin, and nidogen acts as a barrier to drug penetration, reducing therapeutic efficacy and resulting in cancer progression and metastasis (6–10). The presence of regulatory T-cell could be associated with poor survival outcomes (11, 12). Conversely, the enrichment of cytotoxic T cells is associated with prolonged breast cancer survival (13, 14). Biopsy and histopathology remain the gold standard for TME assessment. However, targeted evaluation of individual TME components often requires invasive tissue sampling and component-specific immunohistochemistry, which can limit serial assessment and may not capture whole-tumor heterogeneity. In this context, noninvasive MRI-based radiomic signatures are intended to complement—but not replace—pathology by enabling repeatable, whole-tumor assessment of TME features.

Radiomics refers to the process of extracting high-dimensional data from medical images and objectively describing image characteristics to enable more precise analysis and predictions with carefully designed algorithms (15). Radiomics using magnetic resonance imaging (MRI) data for breast cancer has shown promising results for prognostication, treatment response predicting, and disease characterization (16–18). While most radiomic studies focus on intratumoral regions, recent retrospective studies using peritumoral features have shown utility in breast cancer, such as predicting response to neoadjuvant chemotherapy, human epidermal growth factor receptor 2 (HER2) status, or immune cell infiltration in the TME (18–24).

To test the hypothesis that MRI-based radiomics can noninvasively characterize the TME status of breast cancer, we prospectively evaluated the radiomic characteristics from intratumoral and peritumoral regions on multiparametric MRI—diffusion-weighted imaging (DWI), T2-weighted imaging (T2), and dynamic contrast-enhanced T1-weighted imaging (T1) (25). Our study extends prior work in three ways. First, it is prospective and image biomarker standardization initiative (IBSI)-compliant, with prespecified analyses and adherence to radiomics quality recommendations (25–27). Second, we anchor imaging findings to histology using three-dimensional (3D) volume histology as the reference standard; compared with conventional 2D slides, 3D histology samples have 20–100× thicker tissue volumes and yields more accurate, less biased visualization of TME architecture, including in breast core-needle specimens (28, 29). Third, we assess both principal arms of the TME—ECM and immune cells—and perform head-to-head comparisons across MRI sequences (DWI, T2, dynamic contrast-enhanced T1) and anatomic regions (intratumoral vs. combined intra–peritumoral).

In practice, an ECM-oriented radiomics signature is intended as a complementary adjunct to pathology: It can flag collagen-rich stroma for ECM-modifying approaches or trial referral, suggest immunotherapy stratification, and support serial, noninvasive monitoring during treatment. Model probabilitis are mapped to low/intermediate/high categories using prespecified thresholds and incorporated into a structured report for multidisciplinary review; final diagnostic and therapeutic decisions remain anchored in pathology.

Accordingly, we aimed to determine whether MRI-based radiomics from intratumoral and peritumoral regions can predict ECM and immune components of the TME in invasive breast cancer using DWI, T2, and dynamic contrast-enhanced T1, referenced to 3D histology.

## 2 Methods

### 2.1 Patients

**Figure 1:**
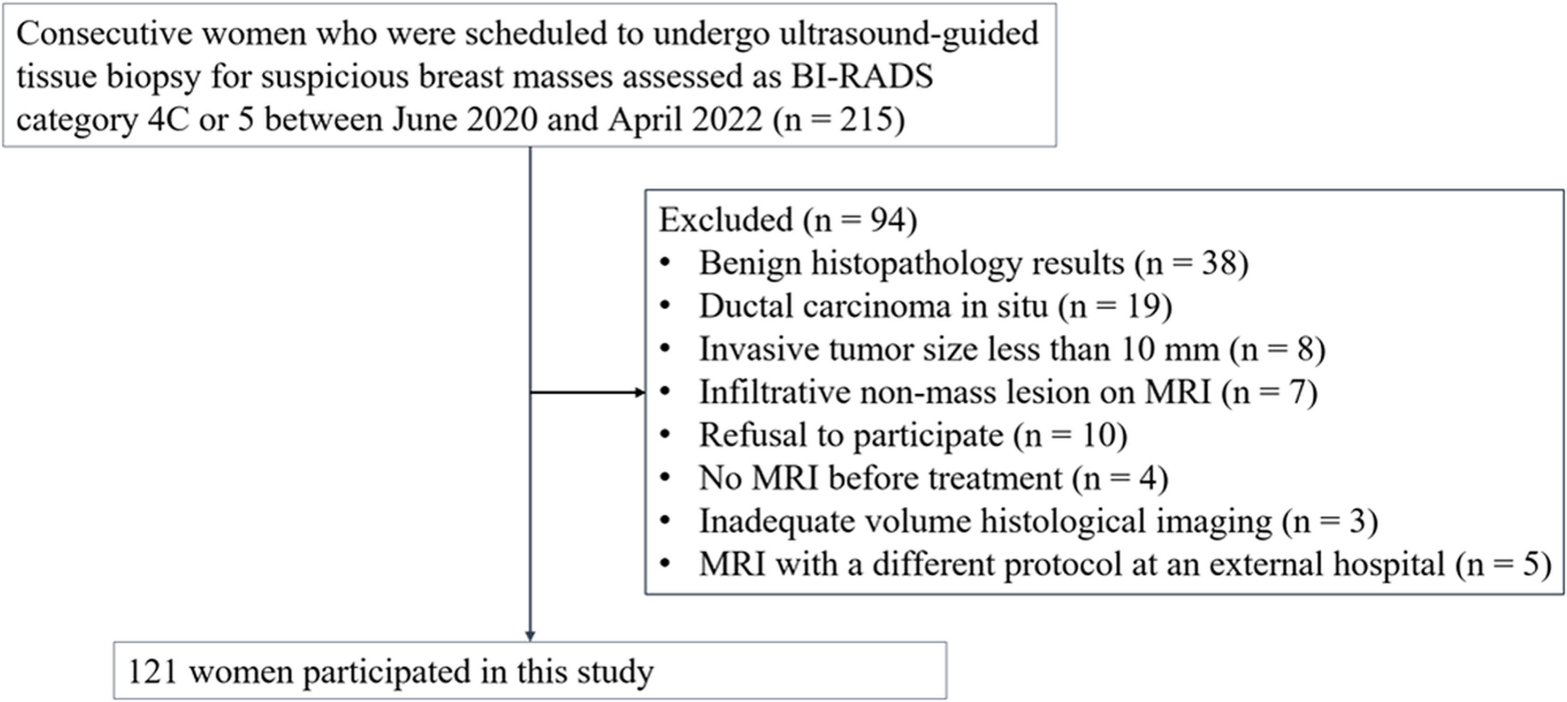
Flowchart of the study participants. 121 participants with invasive breast cancer were included. BI-RADS = Breast Imaging-Reporting and Data System.

This study was approved by our institutional review board and written informed consent was obtained from all participants (Approval Nos. 2020AS0113 and 2021AS0318). This prospective study was registered at clinicaltrials.gov (NCT06095414). Sample size estimation was described in the Supplementary Information. Between June 2020 and April 2022, we enrolled 215 consecutive women who were scheduled to undergo ultrasound-guided tissue biopsy for suspicious breast masses assessed as category 4C or 5 according to the Breast Imaging-Reporting and Data System (30). If a patient had multiple suspicious masses, the most suspicious mass was selected as the representative lesion before biopsy. Ultrasound-guided 14-gauge core-needle biopsy (Bard, Tempe, Arizona, USA) was performed, including the boundary of the tumor. The eligibility criteria were as follows: (a) histologically confirmed invasive breast cancer, (b) participant consent, and (c) MRI performed before treatment. We excluded 94 women for the following reasons: benign histopathology results (n = 38), ductal carcinoma *in situ* (n = 19), invasive tumor size < 10 mm (n = 8), infiltrative nonmass lesion on MRI (n = 7), refusal to participate (n = 10), no MRI before treatment (n = 4), inadequate volume histological imaging (n = 3), and MRI with a different protocol at an external hospital (n = 5). Ultimately, 121 women were included in this study (**Figure 1**).

### 2.2 Histological evaluation of the TME

**Figure 2:**
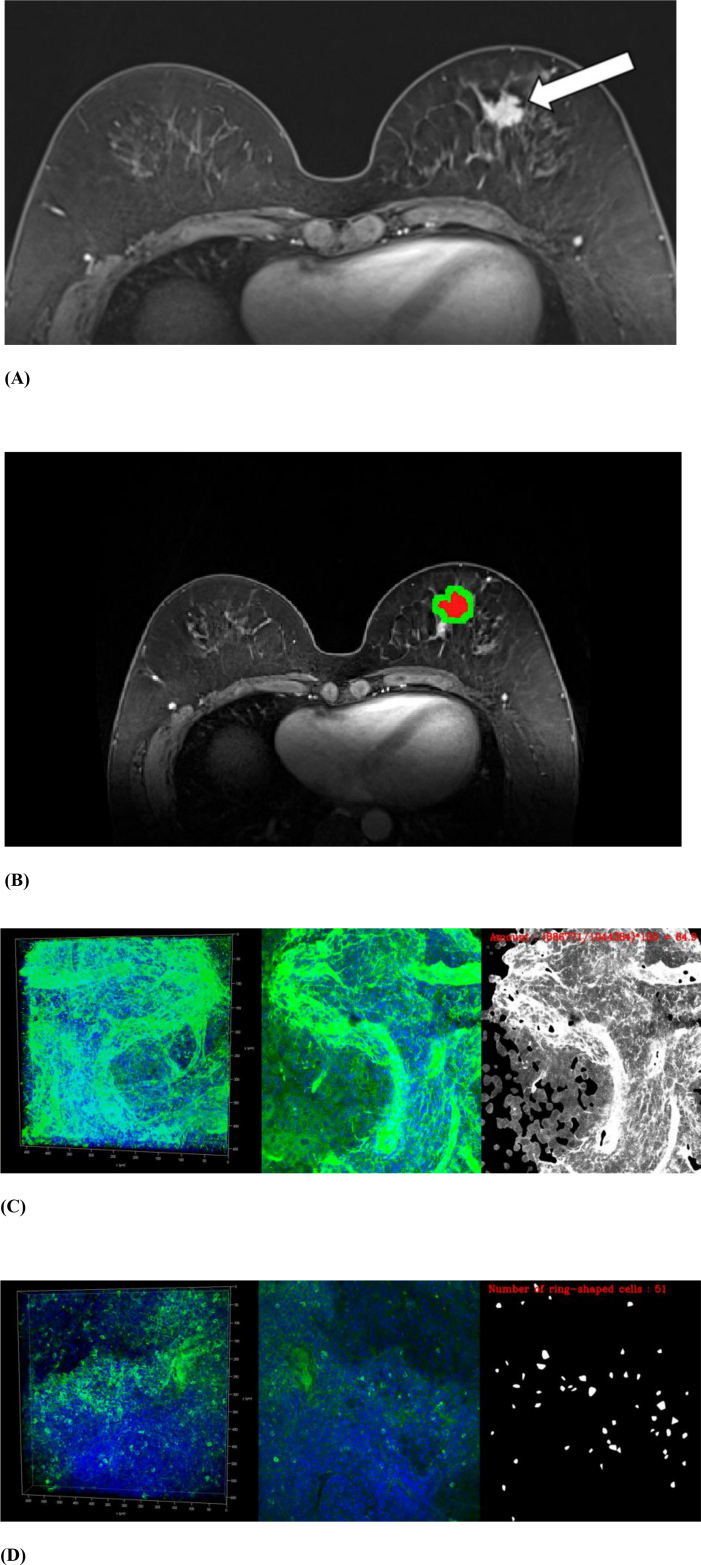
MRI and volume histological images from a 51-year-old woman with triple-negative invasive ductal carcinoma. **(A)** Initial T1-weighted MRI after contrast injection shows an irregularly shaped, heterogeneously enhanced mass (arrow) in the left breast. **(B)** Three-dimensional segmentation of the tumor is performed on the axial image. The intratumoral region is marked in red and the peritumoral region is marked green. The peritumoral region is generated by extending the intratumoral regions with 4 mm thickness in three dimensions and then subtracting the intratumoral region. **(C, D)** Volume histology was performed to evaluate extracellular matrix **(C)** and immune cells **(D)** of tumor microenvironment. To assess the abundance of tumor microenvironmental components, the immunoreactivity of each antibody was quantified by fluorescence imaging. Microscopy images have two channels: blue (channel 1) represents stained cell nuclei and green (channel 2) represents the color for each antibody of tumor microenvironment components. To assess the expression of extracellular matrix components such as collagen **(C)**, the amount of immunopositivity was measured. To assess the abundance of immune cells such as regulatory T cells **(D)**, the number of stained cells is evaluated.

We performed FxClear volume histology, which is an acrylamide-free electrophoretic tissue-clearing protocol with a fast reaction time and high immunoreactivity (31). Volume imaging preserves the structural integrity and allows visualization of the biological architecture in thick tissues and organoids (32, 33). The immunohistochemical reactivity of TME components of each cancer was quantified. Primary antibodies were used to assess the following TME components: collagen type 1 (1:4500, AB34710, Abcam), laminin (1:4500, L9393, Sigma), nidogen-1 (1:9000, NBP1-97701, Novus), FOXP3 (1:600, AB20034, Abcam), and CD8 (1:25, AB75129, Abcam). FOXP3 antibody was used to evaluate regulatory T cells, and CD8 was used to evaluate cytotoxic T cells. The expression of ECM components, including collagen, laminin, and nidogen, was measured as the amount of immunoreactivity. The expression of regulatory and cytotoxic T cells was assessed by counting the number of cells (**Figure 2**). Details of the histological evaluation of the TME are provided in the Supplementary Information. We also reviewed the histological reports to evaluate prognostic factors and subtypes. The histological factors were dichotomized by respective median value unless specified: hormone receptor status including estrogen or progesterone (positive vs. negative), HER2 (positive vs. negative), and Ki67 (high [> 20%] vs. low [≤ 20%]). The molecular subtype was classified into four types according to the St Gallen classification criteria: luminal A, luminal B, HER2-enriched, or triple-negative (34).

### 2.3 MRI acquisition and feature extraction

MRI was performed with the patient in the prone position using a 3T magnet (MAGNETOM Vida; Siemens Healthineers, Erlangen, Germany) with a dedicated 18-channel breast coil. 3D T2, 3D dynamic contrast-enhanced T1, and DWI were obtained in each patient. **Table 1** demonstrates acquisition parameters for MRI. All images were obtained with bilateral axial views and fat saturation. For dynamic contrast-enhanced T1, five postcontrast phases were acquired at 60, 120, 180, 240, and 300 seconds after intravenous contrast administration. DWI was performed using the intravoxel incoherent motion technique.

**Table 1:** Summary of MRI parameters.

Modality	Sequence	TR (ms)	TE (ms)	FOV (mm)	Resolution (mm)	FA (°)	b value (s/mm²)
T1	3D GRASP-VIBE	3.80	1.69	355 × 355	1.01 × 1.01 × 1.00	12	NA
T2	3D SPACE	1000	133	340 × 340	0.33 × 0.33 × 1.00	125	NA
DWI	IVIM	3000	56	340 × 340	1.21 × 1.21 × 4.00	NA	0, 20, 50, 80, 100, 150, 200, 500, 800, 1000, 1500

TR, repetition time, TE , echo time, FOV , field of view, FA , flip angle, T1 , T1-weighted imaging, 3D GRASP-VIBE , 3D golden-angle radial sparse parallel volumetric interpolated breath-hold examination, NA , not applicable, T2, T2-weighted imaging, 3D SPACE , 3D sampling perfection with application-optimized contrasts using different flip angle evolutions, DWI , diffusion-weighted imaging, IVIM , intravoxel incoherent motion.

For radiomics, we analyzed five MRI inputs: T2, DWI (b = 800 s/mm²), precontrast T1, initial-phase postcontrast T1 (60 s), and delayed-phase postcontrast T1 (300 s). In this manuscript, ‘phase’ denotes the two time points (initial and delayed) of the same dynamic contrast-enhanced T1 sequence. Although five DCE time points were acquired (60, 120, 180, 240, 300 s), we pre-specified 60 s and 300 s as the representative phases for radiomics because they correspond to the Breast Imaging-Reporting and Data System (BI-RADS) MRI lexicon windows (initial enhancement vs. delayed persistent/plateau/washout) (35). The initial-phase emphasizes perfusion/permeability, whereas the delayed-phase reflects delayed contrast retention. Intermediate phases (120–240 s) were used only to derive BI-RADS kinetic labels for descriptive reporting and were not included as radiomic inputs to avoid multicollinearity and unnecessary dimensional inflation.

For 3D segmentation of tumors, regions of interest (ROIs) along the entire enhancing tumor margin of cross-sectional area were drawn at axial views of initial-phase contrast-enhanced T1 from top to bottom of each tumor. On precontrast and delayed-phase contrast-enhanced T1, ROIs were drawn on the lesion corresponding to initial-phase contrast-enhanced T1. Because the slice thickness differed for DWI (4 mm) and T2 (3 mm) relative to T1 (1 mm), ROIs for DWI and T2 were drawn separately. Segmentation was performed on axial images by a radiologist (E.S.K. with 5 years of experience in breast MRI) using a semi-automated method with MRIcro software (version 1.40, https://www.nitrc.org/projects/mricro/) under the supervision of a senior radiologist (B.K.S. with 23 years of experience in breast MRI), while blinded to the clinicohistological data except for information about the diagnosis of breast cancer (**Figure 2**). The peritumoral regions were generated by extending the intratumoral regions with 4 mm thickness in 3D. A distance of 4 mm from the tumor was chosen to evaluate the peritumoral area based on previous breast peritumoral radiomics studies (22, 23). In addition, we performed a margin-sensitivity analysis using an 8-mm annulus on the best-performing input from the main analyses to assess robustness.

To assess intra-reader segmentation reproducibility, we performed a stratified random sampling of 50 cases according to tumor-size distribution; each case was re-segmented by the same reader using the identical semi-automated protocol and compared with the original segmentation. Agreement between the two segmentations by the same reader was quantified using the Dice similarity coefficient (DSC) and the Jaccard similarity coefficient (JSC) for volumetric overlap (36).

For feature robustness, isotropic resampling was performed prior to feature extraction. For each of the five MRI inputs, 1618 features were extracted from the intratumoral region and combined intratumoral and peritumoral regions. Thus, a total of 16180 radiomic features were extracted for each cancer. The 1618 features were categorized into four groups: (a) histogram-based first-order statistical features (n = 17), (b) shape and volume features (n = 7), (c) textural features using the gray-level co-occurrence matrix (GLCM) and gray-level run-length matrix (GLRLM) (n = 162), and (d) wavelet-transformed features (n = 1432) (26). We extracted MRI radiomic features in compliance with IBSI (27, 37) using MATLAB R2023b (MathWorks) and the Pyradiomics 3.1.0 library (https://www.radiomics.io/pyradiomics.html) in Python 3.8. The radiomics quality score was 25 out of 36 (69%) (**Supplementary Table 1**) (25). By context, prior oncology radiomics studies have reported a mean radiomics quality score of 9/36 (26%). Details of feature extraction are provided in the **Supplementary Information**.

### 2.4 Feature selection and predictive model construction

After z-score normalization of all features, the dataset was randomly divided into training and testing cohorts in a 7:3 ratio. For each MRI input independently, feature selection was performed using the least absolute shrinkage and selection operator (LASSO) method with fivefold cross-validation to determine the optimal lambda (0.05). The LASSO tool is a statistical method used to select the most relevant features from complex data sets. The LASSO process was iterated 25 times, and features that were selected 20 times during each iteration were considered significant radiomic features. After the process iterations, we identified two sets of five top features from intratumoral features alone and from both intratumoral and peritumoral features and then used these radiomic signatures as predictors in a linear regression analysis (38, 39). Beyond the primary 7:3 split, we performed fivefold cross-validation to assess internally stability and robustness of the predictive models. We report the mean area under the receiver-operating characteristic curve (AUC) and 95% confidence intervals (CI) across folds as the cross-validated performance. Ultimately, we constructed two predictive models: intratumoral-only model based on intratumoral radiomic signatures and a combined intratumoral–peritumoral model combing both intratumoral and peritumoral signatures. To aid interpretability, we visualized the relative contributions of the top 10 selected features as bar plots of standardized coefficients with bootstrap 95% CIs, and assessed redundancy or complementarity using a Pearson correlation heatmap.

### 2.5 Statistical analysis

To assess potential imbalance between the training and testing cohorts, we compared baseline clinicopathological characteristics: age, tumor size, hormone receptor status, HER2 status, Ki67, and molecular subtype. For continuous variables, normality was assessed with the Shapiro–Wilk test, and between-group differences were evaluated using Welch’s *t* test or the Mann–Whitney *U* test, as appropriate. For categorical variables, chi-square or Fisher’s exact tests were applied. We also calculated effect sizes (Cohen’s *d* for continuous variables; absolute differences in proportions for categorical variables), interpreting 0.2, 0.5, and 0.8 as small, medium, and large effects, respectively (40).

In addition, we examined the associations between MRI radiomic features and histological TME components. Model performance was assessed using AUC, accuracy, sensitivity, specificity, positive predictive value, and negative predictive value. To assess the model’s discriminative performance, the AUC was calculated with its 95% CI determined using the bootstrap method, focusing on the 2.5% and 97.5% percentiles of the distribution. The DeLong test was conducted to compare the performance of models using only intratumoral radiomic signatures with models that combine intratumoral and peritumoral radiomic signatures. Additionally, test compared predictive performance across five MRI sequences. A P value < 0.01 (0.05/5), 0.0125 (0.05/4), or 0.025 (0.05/2) from the DeLong test was considered significant, applying Bonferroni correction for multiple comparisons. All reported performance metrics were derived from confusion matrices of our cross-validated predictions.

We evaluated clinical utility using decision-curve analysis. For each model, net benefit was computed across prespecified threshold probabilities (Pt = 0.10–0.40) and compared with treat-none and treat-all strategies. We compared intratumoral-only and combined intratumoral- peritumoral models for two representative endpoints: collagen (ECM) and regulatory T cells (immune cells), using the best performing sequence.

Furthermore, we performed subtype-stratified analyses using the MRI sequence that showed the best overall predictive performance in the main analyses. Within each subtype, intratumoral-only and combined models were evaluated, and pairwise AUC comparisons were conducted using DeLong’s test with Bonferroni correction. To compare performance between subtypes, we used bootstrap resampling (1,000 iterations) to estimate P values and CIs, accounting for unequal and relatively small sample sizes.

**Figure 3:**
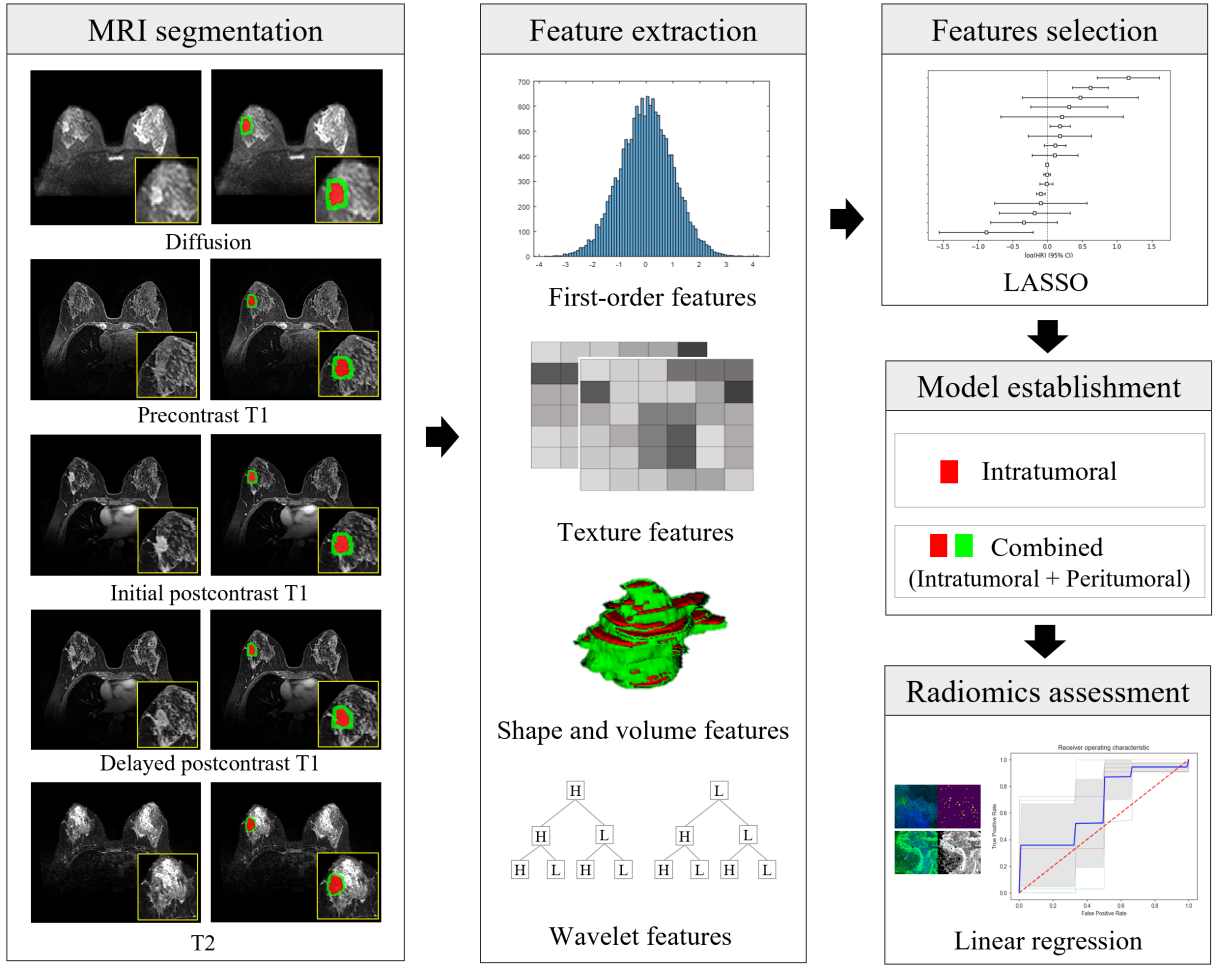
Illustration of the radiomics workflow. After segmentation of the intratumoral (in red) and peritumoral (in green) regions on MRI, a total of 16180 radiomic features (histogram-based first-order statistical features, volume and shape features, texture features, and wavelet-transformed features) were extracted from five MRI sequences of each cancer. Radiomic features were selected, and model building was predicted using the least absolute shrinkage and selection operator (LASSO) and linear regression analysis. The performance of the prediction of each microenvironmental component was obtained using the area under the receiver-operating characteristic curve. Diffusion = diffusion-weighted imaging, initial postcontrast T1 = initial T1-weighted imaging after contrast injection, delayed postcontrast T1 = delayed T1-weighted imaging after contrast injection, T2 = T2-weighted imaging, LASSO = least absolute shrinkage and selector operator.

All statistical metrics were computed using scikit-learn in Python 3.8 (41). The overall design of the study is presented in **Figure 3**. Analysis code is available at [ https://github.com/KUAH-rad/Radiomics-analysis].

## 3 Results

### 3.1 Patient characteristics

We evaluated samples from 121 women (mean age, 54 ± 11 years; age range, 30–83 years) with invasive breast cancer. Of the 121 cancers, 114 were invasive ductal carcinoma (**Figure 2**), five invasive lobular carcinoma, one medullary carcinoma, and one metaplastic carcinoma. **Table 2** summarizes baseline characteristics, and **Supplementary Figure 1** compares the training and testing cohorts. No significant differences were observed for age, tumor size, estrogen receptor, progesterone receptor, hormone receptor, HER2, Ki67, or molecular subtype (all P > 0.05). Effect-size analyses indicated negligible-to-small differences across all variables, with the largest for age (Cohen’s *d* = 0.188) and Ki-67 (*d* = 0.176). Additionally, standardized mean differences for the top 10 radiomic features in the best-performing models—predicting collagen (ECM representative) and regulatory T cells (immune representative)—showed no material imbalances between the training and testing cohorts. (**Supplementary Figure 2**).

**Table 2:** Study participant characteristics.

Characteristics	Value
Age (years)	54 ± 11 (30–83)
Tumor size (mm)	28 ± 18 (10–115)
> 20	64 (53)
≤ 20	57 (47)
Histologic type	
Invasive ductal carcinoma	114 (94)
Invasive lobular carcinoma	5 (4)
Medullary carcinoma	1 (1)
Metaplastic carcinoma	1 (1)
Estrogen receptor	
Positive	102 (84)
Negative	19 (16)
Progesterone receptor	
Positive	99 (82)
Negative	22 (18)
HER2	
Positive	31 (26)
Negative	90 (74)
Ki67	
Positive	52 (43)
Negative	69 (57)
Subtype	
Luminal A	57 (47)
Luminal B	49 (40)
HER2-enriched	4 (3)
Triple-negative	11 (9)

Unless otherwise indicated, data are the number of cancers with percentages in parentheses. Age and tumor size are presented with mean value ± standard deviation and data in parentheses are range. All participants (n = 121) were women. HER2 = human epidermal growth factor receptor 2.

### 3.2 Comparison of TME Prediction Performance between Models using Intratumoral Regions Alone and Models Combining Intratumoral and Peritumoral Regions

**Tables 3**, **4** demonstrate the performance of models using intratumoral radiomics signatures and combing intratumoral and peritumoral radiomic signatures from each MRI sequence. Because model performance ultimately depends on reliable ROI definition, we evaluated intra-reader segmentation reproducibility in 50 randomly selected cases; volumetric overlap was excellent (mean DSC = 0.97 ± 0.01, mean JSC = 0.95 ± 0.02). The radiomic signatures of intratumoral regions included 60 first-order and 65 texture features (62 GLCM features and three GLRLM features), and those of combined regions included 59 first-order, 53 texture features (52 GLCM features and one GLRLM feature), and 13 shape and volume features (**Supplementary Tables 2**, **Supplementary Tables 3**). Among these signatures, the most frequently selected feature for intratumoral regions was the first-order standard deviation and in the combined regions, it was the first-order energy. These two features were found to increase in cancers associated with higher ECM and regulatory T cells, and lower cytotoxic T cells. These conditions, when the TME components were dichotomized based on the median, correlated with a poor prognosis (P < 0.05) (**Supplementary Table 4**).

**Table 3:** Performance of predictive models for tumor microenvironment using intratumoral radiomic signatures.

MRI sequence	Performance	Tumor microenvironmental components				
		Extracellular matrix		Immune cells		
		Collagen	Laminin	Nidogen	Regulatory T Cell	Cytotoxic T Cell
DWI	Accuracy (%)	69 (64, 74)	67 (62, 73)	69 (65, 74)	70 (65, 75)	69 (64, 74)
	Sensitivity (%)	70 (65, 75)	68(62, 74)	70 (65, 75)	70 (66, 75)	72 (67, 76)
	Specificity (%)	70 (63, 76)	63(57, 68)	66 (59, 72)	70 (63, 77)	63 (57, 70)
	PPV (%)	78 (69, 77)	70 (63, 74)	71 (65, 76)	72 (66, 76)	72 (67, 78)
	NPV (%)	46 (38, 51)	49 (44, 53)	51 (45, 57)	50 (45, 54)	49 (40, 55)
	AUC	0.67 (0.63, 0.72)	0.65 (0.60, 0.70)	0.68 (0.64, 0.72)	0.66 (0.63, 0.68)	0.69 (0.66, 0.71)
Precontrast T1	Accuracy (%)	80 (74, 86)	82 (76, 88)	82 (75, 89)	82 (74, 89)	81 (75, 88)
	Sensitivity (%)	80 (74, 86)	83 (79, 87)	85 (77, 92)	83 (78, 88)	83 (76, 90)
	Specificity (%)	68 (63, 73)	78 (70, 85)	77 (73, 81)	71 (65, 77)	63 (58, 68)
	PPV (%)	70 (64, 77)	79 (66, 78)	75 (66, 79)	76 (70, 80)	76 (69, 81)
	NPV (%)	44 (38, 53)	47 (40, 52)	48 (40, 53)	54 (48, 61)	48 (40, 54)
	AUC	0.73 (0.70, 0.76)	0.80 (0.76, 0.83)	0.81 (0.78, 0.84)	0.81 (0.77, 0.85)	0.79 (0.74, 0.83)
Initial postcontrast T1	Accuracy (%)	81 (75, 88)	82 (76, 88)	83 (78, 88)	81 (76, 86)	83 (78, 89)
	Sensitivity (%)	82 (78, 87)	86 (81, 92)	84 (77, 90)	83 (76, 90)	84 (77, 91)
	Specificity (%)	72 (65, 79)	70 (64, 76)	70 (64, 77)	70 (64, 76)	72 (65, 79)
	PPV (%)	79 (69, 82)	79 (68, 83)	80 (74, 85)	79 (68, 83)	80 (74, 85)
	NPV (%)	52 (47, 62)	51 (45, 62)	52 (47, 58)	54 (48, 59)	54 (47, 58)
	AUC	0.80 (0.76, 0.84)	0.81 (0.79, 0.83)	0.81 (0.78, 0.85)	0.80 (0.76, 0.84)	0.82 (0.78, 0.85)
Delayed postcontrast T1	Accuracy (%)	80 (76, 85)	82 (74, 89)	82 (76, 89)	80 (74, 87)	81 (76, 86)
	Sensitivity (%)	82 (74, 89)	83 (77, 88)	83 (76, 90)	81 (75, 87)	82 (78, 86)
	Specificity (%)	70 (64, 77)	77 (72, 82)	70 (65, 75)	70 (65, 75)	69 (63, 74)
	PPV (%)	80 (73, 85)	79 (69, 84)	80 (75, 86)	80 (72, 85)	80 (73, 84)
	NPV (%)	54 (47, 59)	54 (46, 60)	55 (49, 59)	53 (49, 58)	54 (48, 58)
	AUC	0.80 (0.76, 0.83)	0.80 (0.76, 0.85)	0.81 (0.76, 0.86)	0.81 (0.76, 0.86)	0.80 (0.73, 0.86)
T2	Accuracy (%)	81 (77, 85)	79 (73, 86)	78 (72, 84)	79 (73, 86)	80 (75, 85)
	Sensitivity (%)	82 (76, 87)	80 (74, 86)	80 (72, 88)	80 (74, 86)	81 (75, 87)
	Specificity (%)	70 (63, 78)	77 (72, 81)	71 (67, 75)	70 (65, 74)	63 (59, 68)
	PPV (%)	79 (68, 84)	78 (72, 84)	77 (76, 83)	76 (70, 82)	78 (73, 83)
	NPV (%)	50 (44, 56)	52 (48, 55)	54 (46, 58)	53 (48, 58)	51 (48, 56)
	AUC	0.79 (0.75, 0.84)	0.77 (0.71, 0.83)	0.75 (0.71, 0.80)	0.77 (0.72, 0.81)	0.80 (0.76, 0.84)

Data in parentheses are 95% confidence intervals. DWI , diffusion-weighted imaging, T1 , T1-weighted imaging, T2 , T2-weighted imaging, AUC , area under the receiver-operating characteristic curve, PPV , positive predictive value, NPV , negative predictive value.

**Table 4:** Performance of predictive models for tumor microenvironment using intratumoral and peritumoral radiomic signatures.

MRI sequence	Performance	Tumor microenvironmental components				
		Extracellular matrix			Immune cells	
		Collagen	Laminin	Nidogen	Regulatory T Cell	Cytotoxic T Cell
DWI	Accuracy (%)	66 (61,70)	68 (63,74)	70 (64,76)	70 (65,75)	72 (66,77)
	Sensitivity (%)	68 (63,74)	69 (63,75)	73 (66,79)	71 (67,76)	73 (65,77)
	Specificity (%)	61 (56,66)	56 (49,61)	68 (62,73)	69 (63,75)	68 (62,73)
	PPV (%)	70 (66, 73)	71 (68, 79)	73 (68, 78)	75 (70, 79)	74 (68, 77)
	NPV (%)	48 (40, 53)	49 (42, 56)	50 (44, 57)	49 (40, 52)	51 (45, 57)
	AUC	0.64 (0.60, 0.68)	0.66 (0.63, 0.70)	0.73 (0.68, 0.78)	0.69 (0.64, 0.74)	0.71 (0.66, 0.75)
Precontrast T1	Accuracy (%)	81 (75,88)	82 (76,87)	81 (76,86)	83 (78,89)	83 (78,87)
	Sensitivity (%)	80 (73,85)	83 (79,89)	84 (76,89)	86 (80,90)	84 (78,89)
	Specificity (%)	72 (66,79)	78 (72,85)	79 (73,84)	75 (70,79)	79 (74,84)
	PPV (%)	77 (73, 84)	79 (74, 85)	73 (65, 79)	78 (72, 85)	79 (72, 84)
	NPV (%)	45 (38, 49)	48 (43, 52)	48 (43, 54)	55 (47, 60)	49 (42, 54)
	AUC	0.75 (0.71, 0.79)	0.80 (0.75, 0.85)	0.82 (0.77, 0.85)	0.83 (0.79, 0.86)	0.82 (0.76, 0.87)
Initial postcontrast T1	Accuracy (%)	83 (77,86)	83 (76,85)	83 (78,90)	84 (77,90)	84 (81,89)
	Sensitivity (%)	81 (74,89)	84 (76,88)	86 (78,91)	86 (82,90)	82 (78,88)
	Specificity (%)	77 (73,83)	78 (72,84)	79 (73,85)	76 (71,81)	79 (72,82)
	PPV (%)	80 (74, 84)	80 (73, 85)	80 (73, 84)	81 (76, 87)	80 (73, 84)
	NPV (%)	56 (50, 63)	53 (49, 58)	54 (48, 59)	55 (49, 58)	55 (47, 60)
	AUC	0.82 (0.78, 0.87)	0.82 (0.78, 0.86)	0.83 (0.78, 0.89)	0.83 (0.79, 0.88)	0.82 (0.77, 0.89)
Delayed postcontrast T1	Accuracy (%)	83 (78,86)	84 (78,89)	84 (79,90)	84 (78,89)	85 (80,89)
	Sensitivity (%)	80 (77,89)	85 (77,90)	86 (81,90)	85 (79,91)	87 (76,90)
	Specificity (%)	79 (73,85)	71 (67,76)	72 (68,76)	79 (72,86)	76 (68,84)
	PPV (%)	82 (76, 85)	81 (72, 87)	82 (76, 86)	81 (75, 87)	82 (75, 87)
	NPV (%)	56 (50, 62)	56 (49, 61)	58 (49, 63)	56 (48, 61)	55 (43, 60)
	AUC	0.82 (0.79, 0.86)	0.83 (0.78, 0.88)	0.83 (0.76, 0.89)	0.83 (0.78, 0.88)	0.83 (0.79, 0.89)
T2	Accuracy (%)	82 (73,88)	79 (75,84)	80 (75,84)	82 (76,87)	83 (77,88)
	Sensitivity (%)	80 (76,86)	81 (75,88)	81 (76,86)	83 (80,86)	85 (79,88)
	Specificity (%)	74 (68,80)	76 (70,83)	74 (69,80)	74 (69,79)	72 (68,85)
	PPV (%)	79 (72, 85)	77 (70, 84)	79 (72, 84)	76 (70, 82)	75 (68, 80)
	NPV (%)	53 (48, 58)	52 (47, 58)	50 (42, 58)	45 (39, 49)	56 (49, 60)
	AUC	0.79 (0.77, 0.85)	0.79 (0.74, 0.85)	0.78 (0.72, 0.84)	0.82 (0.77, 0.87)	0.82 (0.78, 0.88)

Data in parentheses are 95% confidence intervals. DWI , diffusion-weighted imaging, T1 , T1-weighted imaging, T2 , T2-weighted imaging, AUC, area under the receiver-operating characteristic curve, PPV = positive predictive value, NPV , negative predictive value.

**Table 5** shows the comparison of AUCs between two models using intratumoral radiomic signatures alone and combined intratumoral and peritumoral radiomic signatures to predict TME. Models using combined radiomic signatures showed significant improvements in predicting laminin on delayed postcontrast T1 (AUC [95% CI] for combined model vs. intratumoral model, 0.83 [0.78, 0.88] vs. 0.80 [0.76, 0.85]), nidogen on DWI (0.73 [0.68, 0.78] vs. 0.68 [0.64, 0.72]), regulatory T cells on DWI (0.69 [0.64, 0.74] vs. 0.66 [0.63, 0.69]), regulatory T cells on T2 (0.82 [0.77, 0.87] vs. 0.77 [0.72, 0.81]), and cytotoxic T cells on precontrast T1 (0.82 [0.76, 0.87] vs. 0.79 [0.74, 0.83]) (P < 0.01).

**Table 5:** Comparison of AUCs between two models using intratumoral radiomic signatures and combined intratumoral and peritumoral radiomic signatures to predict tumor microenvironmental components.

MRI sequence	Tumor microenvironmental components				
	Extracellular matrix			Immune cells	
	Collagen	Laminin	Nidogen	Regulatory T cell	Cytotoxic T cell
DWI	0.023	0.042	0.008	0.009	0.012
Precontrast T1	0.024	0.057	0.034	0.017	0.009
Initial postcontrast T1	0.016	0.036	0.018	0.013	0.034
Delayed postcontrast T1	0.016	0.009	0.017	0.015	0.012
T2	0.051	0.038	0.016	0.007	0.014

Data are given as P value by DeLong’s test. A P value < 0.01 (0.05/5) is considered significant, applying Bonferroni correction for multiple comparisons. DWI , diffusion-weighted imaging, T1 , T1-weighted imaging, T2 , T2-weighted imaging, AUC , area under the receiver-operating characteristic curve.

The bar plot of standardized coefficients highlighted a subset of top features with the greatest contributions (**Supplementary Figure 3**). The Pearson correlation heatmap demonstrated clustered groups of highly correlated features and low inter-feature correlations (**Supplementary Figure 4**).

### 3.3 Comparison of TME prediction performance according to MRI sequences

When comparing performance between five MRI sequences in models using combined intratumoral and peritumoral radiomic signatures, delayed postcontrast T1 yielded the highest AUCs (AUC [95% CI] for collagen 0.82 [0.79, 0.86], laminin 0.83 [0.78, 0.88], nidogen 0.83 [0.76, 0.89], regulatory T cells 0.83 [0.78, 0.88], and cytotoxic T cells 0.83 [0.79, 0.89]) (**Table 4**). The AUCs of delayed postcontrast T1 were significantly superior to those of DWI in predicting all the TME components, precontrast T1 in predicting collagen, and T2 in predicting nidogen (P < 0.0125) (**Table 6**). However, there was no difference in AUCs between delayed postcontrast T1 and initial postcontrast T1 for all TME components (*P ≥* 0.0125). The combination of initial postcontrast T1 and delayed postcontrast T1 did not show a significant improvement over using either initial or delayed postcontrast T1 alone (*P ≥* 0.025) (**Supplementary Table 5**).

**Table 6:** Comparison of AUCs between different MRI sequences in models using combined intratumoral and peritumoral regions to predict tumor microenvironmental components.

MRI sequence	Tumor microenvironmental components					
	Extracellular matrix				Immune cells	
	Collagen	Laminin	Nidogen	Regulatory T cell		Cytotoxic T cell
Delayed postcontrast T1 vs. DWI	0.002	0.001	0.006	0.003		0.005
Delayed postcontrast T1 vs. precontrast T1	0.011	0.014	0.042	0.059		0.047
Delayed postcontrast T1 vs. initial postcontrast T1	0.047	0.038	0.074	0.075		0.068
Delayed postcontrast T1 vs. T2	0.026	0.017	0.011	0.019		0.039

Data are given as P values by DeLong’s test. A P value < 0.0125 (0.05/4) is considered significant, applying Bonferroni correction for multiple comparisons. DWI , diffusion-weighted imaging, T1 , T1-weighted imaging, T2 , T2-weighted imaging, AUC , area under the receiver-operating characteristic curve.

**Figure 4:**
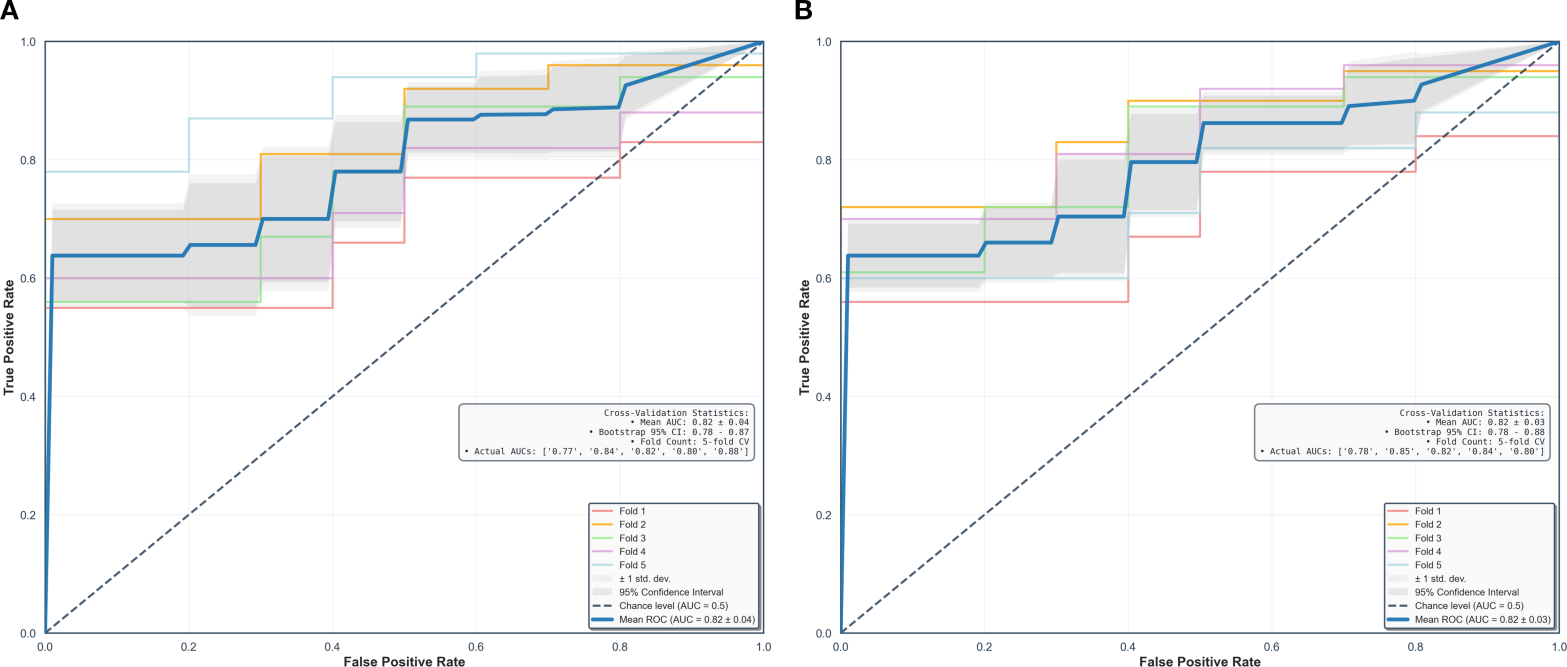
Performance in combined models using intratumoral and peritumoral regions on initial postcontrast T1-weighted images. The AUCs (95% confidence interval) of the overall performance in predicting ECM **(A)** and immune cell **(B)** abundance are 0.82 (0.78, 0.87) and 0.82 (0.78, 0.88). AUC = the area under the receiver-operating characteristic curve, CI = confidence interval.

**Figure 5:**
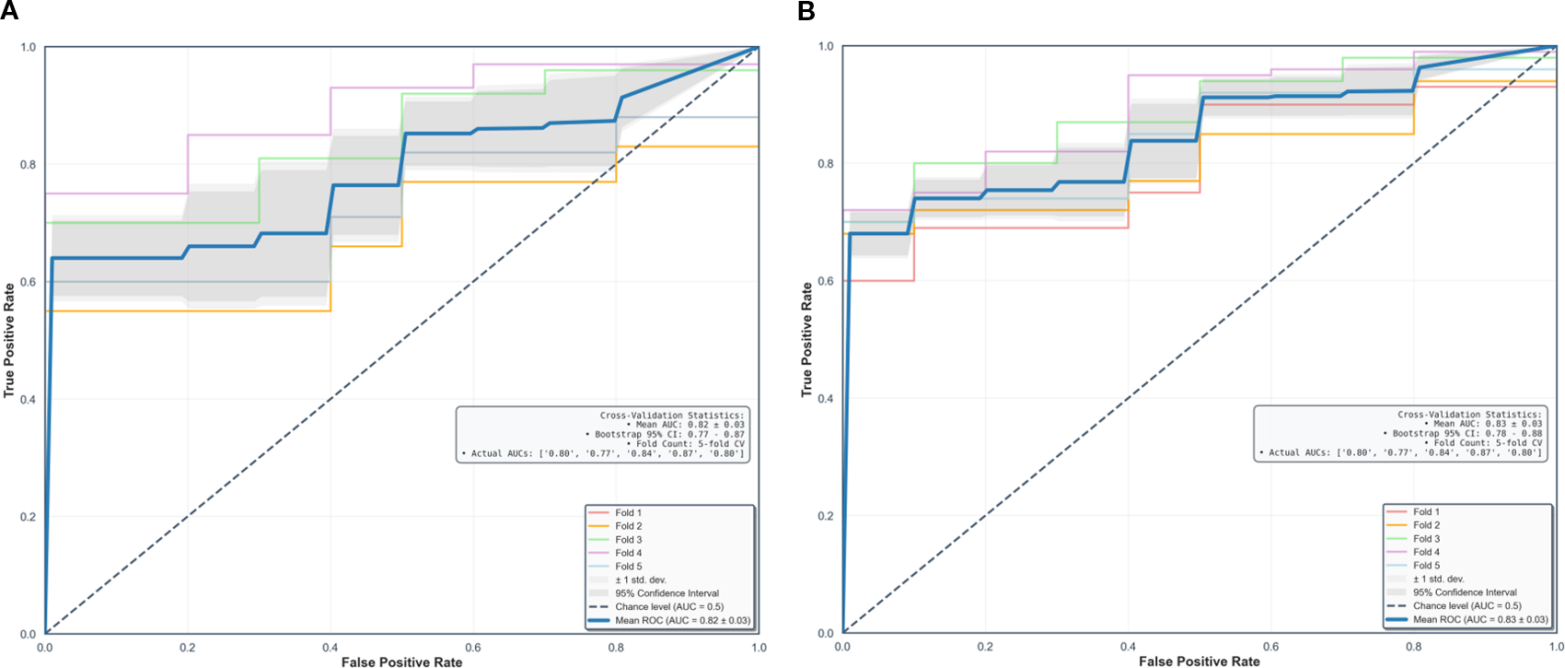
Performance in combined models using intratumoral and peritumoral regions on delayed postcontrast T1-weighted images. The AUCs [95% confidence interval] of the overall performance in predicting ECM **(A)** and immune cell **(B)** abundance are 0.82 (0.77, 0.87) and 0.83 (0.78, 0.88). AUC = the area under the receiver-operating characteristic curve, CI = confidence interval.

Using fivefold cross-validation, the AUCs of the overall performance in predicting the abundance of ECM and immune cells using combined features were 0.82 [0.78, 0.87] and 0.82 [0.78, 0.88] on initial postcontrast T1 and 0.82 [0.77, 0.87] (**Figure 4**) and 0.83 [0.78. 0.88] on delayed postcontrast T1, respectively (**Figure 5**). As shown in these figures, the “Actual AUCs” listed in the figure panels represent the values obtained from each fold of the fivefold cross-validation. **Table 7** shows radiomic signatures for predicting overall ECM and immune cell abundance on initial or delayed postcontrast T1. Nineteen (95%) out of 20 radiomic signatures were first-order and GLCM texture features.

**Table 7:** Radiomic signatures from combined intratumoral and peritumoral regions to predict overall extracellular matrix and immune cell abundance on postcontrast T1.

MRI sequence	Tumor microenvironmental components	
	Extracellular matrix	Immune cells
Initial postcontrast T1	First-order_Mean	First-order_Skewness
	First-order_Kurtosis	First-order_Std
	First-order_Std	GLCM_Entropy_Std
	First-order_Range	GLCM_Contrast
	GLCM_Contrast	First-order_Mean
Delayed postcontrast T1	First-order_Sum	First-order_Energy
	First-order_Energy	First-order_Mean
	First-order_Median	Shape and volume_Sphericity
	GLCM_Cluster Shade	First-order_Energy
	First-order_Mean	First-order_Kurtosis

The radiomic feature name is described by group_discriptor_statistic. T1 , T1-weighted imaging, GLCM , gray-level co-occurrence matrix, Std standard deviation.

Decision-curve analysis demonstrated that the combined intratumoral-peritumoral models provided consistently greater net benefit than intratumoral-only models across the clinically relevant threshold range (P*_t_* = 0.10-0.40) (**Supplementary Figure 5**). For collagen, the combined model outperformed the intratumoral-only model across all threshold, and both exceeded the treat-all and treat-none strategies. Similarly, for regulatory T cells, the combined model consistently yielded higher net benefit than the intratumoral-only model within the same threshold range. In the combined models on delayed-phase postcontrast T1, we performed correlation analyses between the top radiomic features and quantitative histology metrics, collagen (ECM) and regulatory T cells (immune cells). GLCM contrast was significantly correlated with collagen proportion, and first-order standard deviation was correlated with regulatory T-cell density (both P < 0.001) (**Supplementary Figure 6**).

In subtype-stratified analyses using the delayed postcontrast T1-weighted sequence, luminal A cancers showed AUCs of 0.59-0.61 for intratumoral-only models and 0.64-0.67 for combined intratumoral–peritumoral models (**Supplementary Tables 6**, **Supplementary Tables 7**). Luminal B cancers showed AUCs of 0.59-0.60 for intratumoral-only models and 0.62-0.65 for combined models. Triple-negative breast cancer showed AUCs of 0.56-0.59 for intratumoral-only models and 0.58–0.64 for combined models. Across subtypes, combined models tended to outperform intratumoral-only models for TME prediction, but differences did not reach statistical significance after correction for multiple comparisons (P > 0.016) (**Supplementary Table 8**). In the combined models, ECM prediction for luminal A cancers was higher than for triple-negative cancers (collagen P = 0.031; nidogen P = 0.28); however, these pairwise differences did not meet the Bonferroni-corrected significance threshold (P = 0.025 for two comparisons, 0.05/2) (**Supplementary Table 9**). HER2-enriched tumors (n = 4) were excluded from statistical testing because such a limited cohort renders receiver-operating characteristic curve estimation unstable and prevents reliable inference.

Additionally, we performed a peritumoral margin-sensitivity analysis (4 mm vs. 8 mm) on delayed postcontrast T1. the 8 mm annulus yielded lower AUCs, supporting 4 mm as the primary peritumoral margin (**Supplementary Table 10**).

## 4 Discussion

This prospective study highlights the potential of MRI-based radiomics to noninvasively predict TME components, including the ECM and immune cells, in invasive breast cancer. Radiomic features were extracted from both intratumoral and peritumoral regions across DWI, T2, and dynamic contrast-enhanced T1. Combined models incorporating both intratumoral and peritumoral features outperformed models using intratumoral features alone, with initial or delayed postcontrast T1 showing the best predictive performance (AUC 0.82–0.83).

Most previous breast radiomic studies have focused on intratumoral features and overlooked information in the peritumoral regions (16, 17). However, a growing body of evidence indicates that characteristics of the peritumoral regions can provide information about changes in the TME (24, 42–45). Braman et al. (45) found that incorporating both intratumoral and peritumoral MRI radiomic features improved the AUC for identifying HER2-enriched subtypes (0.89 vs. 0.76) and predicting the pathological response to anti-HER2 therapy (0.80 vs. 0.66). They also reported a significant association between peritumoral radiomic features and the density of tumor-infiltrating lymphocytes. Similarly, a recent retrospective study by Qian et al. (24) demonstrated that a combined model showed higher performance in predicting the level of immune cell infiltration of M2 macrophage than an intratumoral model (AUCs of training and testing cohorts; 0.84 and 0.74 vs. 0.66 and 0.68). They utilized the arterial phase of postcontrast T1 for radiomic analysis. In our study, both initial and delayed postcontrast T1 showed superior performance in predicting the TME, with no significant difference between two sequences. The improvement in predicting the immune cell TME with combined models using both intra- and peritumoral radiomic features, rather than only intratumoral features, aligned with our study findings.

In the present study, the most frequently selected radiomic features were first-order (notably standard deviation and energy) and GLCM textures. Increases in standard deviation and decreases in energy indicate reduced histogram-based uniformity and GLCM contrast captures local intensity differences consistent with textural heterogeneity (46). GLCM contrast was positively correlated with collagen proportion, and first-order standard deviation was positively correlated with regulatory T-cell density (both P < 0.05). These feature–histology associations suggest that radiomic heterogeneity reflects stromal organization (collagen) and the immune milieu (regulatory T cells), reinforcing the biological plausibility of the learned signatures.

Among MRI sequences, DWI-based models showed inferior performance compared to T1- or T2-based models, likely due to DWI’s lower resolution and fewer segmented pixels per lesion (47). Enhancing the resolution of DWI or utilizing super-resolution apparent diffusion coefficient maps extracted from DWI may improve DWI-based radiomics performance (47, 48). In this study, delayed postcontrast T1 presented the highest predictive performance. Mechanistically, dynamic postcontrast imaging samples contrast-agent kinetics: the initial-phase reflects microvascular perfusion/permeability (wash-in), whereas the delayed-phase captures late contrast retention influenced by interstitial transport and stromal organization (49, 50). A collagen-rich, fibrotic ECM can increase interstitial resistance and prolong late retention, while immunosuppressive/angiogenic milieus can produce heterogeneous early enhancement; these vascular–stromal interactions are expressed as peritumoral texture heterogeneity, plausibly explaining the superior performance of delayed T1 over static contrasts. The superiority of delayed postcontrast T1 may reflect the reduced influence of early angiogenic perfusion/permeability effects, while persistent contrast retention preserves lesion-to-stroma conspicuity for visualizing ECM architecture. Reig et al. (51) reported that the usefulness of delayed postcontrast T1 in identifying residual tumors after neoadjuvant chemotherapy. Residual lesions may show late enhancement due to the anti-angiogenic effects of chemotherapy. Further studies are warranted to validate the performance of various MRI sequences in predicting each TME component in a larger number of patients for tailored treatment and predicting response to different treatment modalities.

Although improvements in discrimination between combined intratumoral–peritumoral and intratumoral-only models were modest, decision-curve analysis provided complementary evidence of clinical utility. For both collagen and regulatory T cells, the combined models showed consistently higher net benefit across clinically plausible thresholds, indicating that peritumoral information enhances risk stratification. These findings underscore the potential clinical value of incorporating tumor-adjacent radiomic features beyond the tumor boundary. Our margin-sensitivity experiment further contextualizes this finding. On delayed postcontrast T1, expanding the peritumoral annulus from 4 mm to 8 mm reduced performance, consistent with signal dilution from inclusion of non–tumor-proximal tissue. These results support 4 mm as an appropriate operating margin for the main analyses.

Strengths of our study include the prospective, histology-anchored design; adherence to IBSI recommendations; and a comparatively high radiomics quality score (25/36) relative to prior oncology radiomics reports (25). In addition, intra-reader segmentation reproducibility was excellent, with DSC and JSC values exceeding 0.95, reinforcing the robustness of the semi-automated segmentation process and supporting the reliability of radiomic feature extraction. For clinical integration, we specify how the ECM- or immune cell–oriented radiomics signatures would be implemented as adjuncts to pathology: standardized pretreatment MRI; tumor segmentation on early postcontrast T1 with a 4 mm peritumoral annulus and mask propagation to the delayed-phase; feature extraction and model inference prioritizing delayed postcontrast T1 with intra- and peritumoral features; and probabilities mapped as low, intermediate, or high using decision-curve–informed thresholds and presented in a structured report addendum for multidisciplinary review. Deployment proceeds from silent to assisted mode with ongoing calibration and drift monitoring; final management decisions remain in conjunction with pathology. Future work will prioritize multicenter, multi-scanner external validation with protocol harmonization to ensure generalizability. Methodologically, we will evaluate delta-radiomics on dynamic MRI—both phase-based (early to delayed) and longitudinal changes during therapy—to test sensitivity to TME remodeling. We will also pursue multi-omics integration (radiomics with genomic signatures of ECM and immune activity) to improve biological interpretability and predictive performance. Clinically, we will study neoadjuvant cohorts with serial TME assessment to support early response adaptation and treatment guidance. Finally, we will incorporate automated segmentation, rigorous calibration/decision-curve analyses, and stepwise prospective deployment (silent to assisted) to assess clinical utility and workflow impact.

Limitations include the following. First, this was a single-center study on a single 3T system with a fixed protocol; we did not assess multi-scanner variability or validate on an external dataset, which may limit generalizability. Second, although statistically justified, the sample size is relatively small for high-dimensional radiomic modeling, increasing the risk of overfitting. These limitations underscore the need for larger, multicenter cohorts with multi-scanner acquisition and external validation, which we plan to pursue. Third, we did not perform automated segmentation. To minimize issue with lesion selection, semiautomatic segmentation was performed by the breast specialist under the supervision of the senior breast specialist. In addition, intra-reader segmentation reproducibility was excellent, with DSC and JSC values exceeding 0.95, reinforcing the robustness of the semi-automated segmentation process and supporting the reliability of radiomic feature extraction. Future studies incorporating fully automated segmentation pipelines to further improve robustness and reproducibility are warranted. Fourth, patients with invasive tumors smaller than 10 mm were excluded. Because the slice thickness of T2 (3 mm) and DWI (4 mm) was larger than that of T1 (1 mm), partial volume effects and misregistration across sequences could have compromised segmentation accuracy and reproducibility in very small lesions. Moreover, the restricted voxel counts in such tumors frequently led to unstable or infeasible radiomics feature extraction. This exclusion criterion may have limited the applicability of our findings to subcentimeter tumors. Fifth, in subtype-stratified analyses, discriminative performance was modest across subtypes. Although combined intra–peritumoral models tended to outperform intratumoral-only models for TME prediction, the gains were not significant. This likely reflects limited sensitivity to subtype-specific biology under the current sample sizes: the HER2-enriched subgroup was excluded from statistical testing (n = 4), and the remaining subgroups were small and imbalanced, leading to wide CIs and reduced power. Larger, balanced, multicenter cohorts with multi-scanner acquisition and multi-reader assessment are needed to validate TME prediction at the subtype level and to support clinical implementation.

## 5 Conclusion

MRI-based radiomic models that incorporate both intratumoral and peritumoral features demonstrated superior performance over intratumoral-only models in characterizing the TME in invasive breast cancer. Among the imaging sequences, contrast-enhanced T1—particularly the delayed-phase—showed the highest predictive accuracy for ECM and immune cell components. These findings suggest that radiomics has the potential to serve as a noninvasive biomarker for assessing TME profiles, including immunosuppressive and ECM-rich phenotypes associated with poor prognosis. With further validation in large, multicenter cohorts, this approach may contribute to personalized treatment planning and response prediction in breast cancer.

## Data Availability

The original contributions presented in the study are included in the article/**Supplementary Material**. Further inquiries can be directed to the corresponding authors.
